# Development of an *in vitro* aggregation assay for long synthetic polypeptide, amyloidogenic gelsolin fragment AGelD187N 173–242

**DOI:** 10.1371/journal.pone.0290179

**Published:** 2023-08-17

**Authors:** Laura Leimu, Oskar Haavisto, Victor Nesati, Patrik Holm, Antti Haapalinna, Rune Salbo, Ullamari Pesonen

**Affiliations:** 1 R&D, Orion Pharma, Orion Corporation, Turku, Finland; 2 Faculty of Medicine, Institute of Biomedicine, University of Turku, Turku, Finland; Instituto Mexicano del Seguro Social, MEXICO

## Abstract

Aggregation of the gelsolin protein fragment is the hallmark of the hereditary systemic disease gelsolin amyloidosis. As with other protein misfolding diseases, there is an urgent need for efficient disease-modifying treatment for gelsolin amyloidosis. The formation of amyloids can be reproduced by incubating the disease-causing amyloidogenic 8 kDa polypeptide, 70-residue gelsolin protein fragment, AGelD187N 173–242, *in vitro* and monitoring the process by thioflavin T dye. However, for screening of potential aggregation inhibitors, the required protein amounts are large and the biotechnological production of amyloidogenic proteins has many challenges. Conversely, use of shorter synthetic regions of AGelD187N 173–242 does not mimic the *in vivo* aggregation kinetics of full-length fragment as they have different aggregation propensity. In this study, we present an *in vitro* aggregation assay for full-length AGelD187N 173–242 that has been produced by solid-phase chemical synthesis and after that monomerized carefully. Chemical synthesis allows us to produce high quantities of full-length fragment efficiently and at low cost. We demonstrate that the generated aggregates are fibrillar in nature and how the purity, terminal modification, initial aggregates and seeding affect the aggregation kinetics of a synthetic gelsolin fragment. We also present sufficient quality criteria for the initial monomerized synthetic polypeptide.

## Introduction

Gelsolin amyloidosis (AGel amyloidosis) is a hereditary systemic protein misfolding disease that is caused by mutations in the gelsolin gene. The functional mutations predispose the secreted plasma gelsolin to aberrant proteolytic cleavages. The first identified and best-known mutation is the D187N variant, the so-called Finnish variant [1]. This mutation compromises the stability of the protein and leads to the formation of amyloidogenic fragments. The main product is the 8 kDa fragment comprising of 70 amino acid residues, AGelD187N 173–242. The gelsolin amyloidosis patients with the Finnish mutation will experience a triad of ophthalmological, neurological and dermal symptoms as a cause of gelsolin fragment accumulation in the basement membrane of the skin, blood vessel walls, eyes and peripheral nervous system [2]. This will cause severe disease burden starting from young adulthood [3]. As currently the only treatments available are alleviation of symptoms and support care, there is a high unmet medical need for disease modifying treatments.

When designing possible therapeutic interventions that would inhibit the aggregation, imitation of the aggregation process of amyloidogenic peptides or proteins is crucial. Although *in vitro* aggregation assays are much simplified compared to the *in vivo* situation [4], they are helpful in detecting potential anti-aggregational hit compounds. To execute the aggregation process on a laboratory time scale and in a high-throughput fashion, the amyloidogenic protein is needed in large quantities. The biotechnological production of amyloidogenic proteins has many challenges due to their intrinsic hydrophobicity [5]. If amyloid proteins are expressed and purified in bacterial cells without fusion partners, they easily form insoluble inclusion bodies. The denaturation and renaturation process of inclusion bodies is cumbersome and time-consuming. Residual chaotropic reagents will greatly affect the aggregation of amyloid proteins. If a fusion partner is used for soluble expression and easier purification of an amyloid protein, the fusion partner must be removed to obtain the authentic protein. This will lead in low yield and purity without an additional purification step. All purification and concentration steps are complicated by the fact that amyloid proteins are particularly prone to adsorb on the filters used in the processes.

The solid-phase chemical synthesis is efficient and inexpensive method to produce large amounts of peptides [6]. However, the maximum size of polypeptide that can be reliably made in a good yield as a homogenous molecular species has been limited to around 50 amino acids [7–10]. The longer the peptide, the greater the risk for impurities. In addition to this, protein aggregation studies are especially sensitive to any variation in the initial quality of the protein [11,12]. With regards to the gelsolin fragment AGelD187N 173–242, all previous aggregation studies have been made with recombinant material produced in *Eschericia coli* [13–15]. These studies have generated valuable understanding concerning the aggregation behavior of AGelD187N 173–242, but their focus has not been from the practical aspect of drug discovery, and they have not addressed the steady supply of large amounts of amyloidogenic protein. Some aggregation studies have also been performed with recombinantly produced isolated G2 domain of gelsolin [16,17]. The relevance of these studies is questionable because the amyloidogenic fragment is still buried inside the folded domain. With regards to synthetic material, only short stretches of AGelD187N 173–242 have been used in aggregation studies [16,18–20]. These short peptides are believed to represent the amyloidogenic region of the fragment. However, the amyloid core of patient-derived AGel fibrils has not been defined by any structural analyses. Also, in light of the latest information, the role of flanking regions around the amyloid core is much more important in the fibril formation than previously thought [21]. The aggregation propensity of short peptides is different compared to the full-length fragment [22] and there is a high risk that the fibrils formed from short peptides are not *in vivo* relevant. Use of full-length AGelD187N 173–242 would be more appropriate when screening inhibitors also because important binding epitopes for inhibitors can be missing from the short peptides.

In this study, we report that synthetic 70-residue AGelD187N 173–242 polypeptide is able to form fibrils typical of amyloid diseases *in vitro*. By applying stringent monomerization of polypeptide by gel filtration chromatography and following the aggregation using thioflavin T (ThT) as the reporter, we were able to elicit the typical sigmoidal aggregation curve for synthetic AGelD187N 173–242 reported for recombinant gelsolin fragment [13–15] and other amyloidogenic peptides [23,24]. By applying seeding, we were able to follow the aggregation of synthetic AGelD187N 173–242 in efficient and reproducible manner that allows the screening of possible inhibitors. To our knowledge, this is the first time that this long amyloidogenic polypeptide made by solid phase chemical synthesis has been successfully used for aggregation studies.

## Materials and methods

### Polypeptides

**Fig 1:**
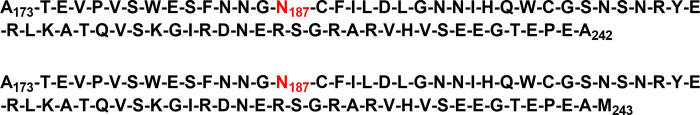
The amino acid sequences for the polypeptides used in the study. The polypeptide sequence of the 70-residue fragment ending on alanine, AGelD187N 173–242 (upper sequence) and the 71-residue fragment ending on methionine, AGelD187N 173–243 (lower sequence). Amino acid numbering is based on the full-length human plasma gelsolin (UniProtKB P06396-1) without the signal peptide (amino acids 1–27). The mutation sites are marked in red.

All synthetic polypeptides used in this study were supplied by Caslo ApS, Denmark. The full-length human plasma gelsolin (UniProtKB P06396-1) amino acid numbers, terminal modifications, reversed-phase-high performance liquid chromatography (RP-HPLC) purities and batch sizes are listed in Table 1. The used nomenclature is based on the recommendations by the International Society of Amyloidosis (ISA) nomenclature committee [25,26]. The name AGelD187N in the text refers to all three polypeptides used in the study and the name AGelD187N 173–242 is used interchangeably to refer to AGelD187N 173–242 (95%), which was mainly used in this study. The amino acid sequences of AGelD187N 173–242 and AGelD187N 173–243 are presented in Fig 1.

**Table 1:** Synthetic polypeptides used in the study.

Polypeptide	Amino acid numbers	Terminalmodifications	RP-HPLC purity	Batch size
**AGelD187N 173–242 (95%)**	**173–242**	**Amidated C-terminus**	**95%**	**50 mg**
AGelD187N 173–242 (90%)	173–242	Amidated C-terminus	90%	50 mg
Ac-AGelD187N 173–243 (95%)	173–243	N-terminus acetylated, amidated C-terminus	95%	5 mg

AGelD187N 173–242 (95%) is highlighted since it was used in most parts of the study. The shorter name AGelD187N 173–242, used in the text, refers to this polypeptide. AGelD187N in the text refers to all three polypeptides.

The polypeptides were prepared by manual solid-phase peptide synthesis, with Fmoc-Ala-Rink Amide resin as the solid support. All the chemicals were purchased from main chemical suppliers and used without further purification. Fmoc groups for N^α^-protection were cleaved by 8 min treatment with 20% piperidine in dimethylformamide (DMF) followed by the second treatment with the same reagent for 10 min. After the Fmoc cleavage, the peptide-resin was washed with DMF (× 6). The next residue was then incorporated with the HATU/DIPEA coupling protocol. After gentle agitation (1 hr) and washing with DMF (× 6), part of the peptide-resin was subjected to Kaiser test. On completion of the assembly, the peptide-resin was successively washed with DMF (× 3), dichloromethane (× 4) and then dried *in vacuo*. Complete deprotection was achieved in a trifluoroacetic acid cleavage cocktail with scavengers in 3 hrs at 30°C. Following the cleavage reaction, the mixture was precipitated in cold ether and dried *in vacuo*. The crude was purified by preparative RP-HPLC using C18 50 mm x 250 mm column (100 Å pore size, 10 μM particle size, Agilent). The eluate was collected and then lyophilized. The overall yield was 6%. The purity of the polypeptides was assessed by analytical RP-HPLC using C18 4.6 mm x 250 mm column (Kromasil, 5 μm, Agilent) and the correct sequence was verified by electrospray ionization (ESI) mass spectrometry (S1 File).

### Isolation of monomeric polypeptide

AGelD187N polypeptides were dissolved in 6 M guanidine hydrochloride (Sigma-Aldrich) in PBS (Gibco) and sonicated in water bath sonicator for half an hour to break up any aggregates or oligomers. The polypeptide solution was then centrifuged for 15 min at 20 000 rpm and loaded into PBS pre-equilibrated Superdex 75 Increase 10/300 GL gel filtration column (GE Healthcare) to buffer exchange and remove any aggregated species. From the monomer peak, three or more main fractions (usually 3 x 250 μl) were collected on ice and UV-Vis spectrophotometric measurement (DS-11, Denovix) was used to determine the polypeptide concentration using an extinction coefficient of 15,780 M^-1^cm^-1^. In more stringent monomerization protocol, only one fraction (250 μl) from the middle of the main peak was collected during monomerization. The monomerized polypeptide was immediately diluted to 30 μM with PBS and used in the aggregation studies.

### Characterization of monomerized polypeptide by SEC

The monomerized polypeptide was characterized by analytical size exclusion chromatography (SEC). Analytical SEC was performed on the monomerized AGelD187N polypeptide by using Advance Bio SEC 4.6 mm x 300 mm column (300 Å pore size, 2.7 μM particle size, Agilent) combined with Advance Bio SEC 4.6 mm x 50 mm guard column (300 Å pore size, 2.7 μM particle size, Agilent). Isocratic elution was performed with a mobile phase (150 mM sodium phosphate buffer, pH 7.0) flow rate of 0.35 ml/min. The column was connected to an HPLC system (1200 Infinity, Agilent) sequentially with a diode array detector (DAD WR, 1200 Infinity, Agilent).

### Thioflavin T aggregation assay

Stock solutions of 500 μM thioflavin T (ThT) (Sigma-Aldrich) and 150 μM heparin (Mw = 17–19 kDa, Sigma-Aldrich) were prepared by dissolving the dry powders in PBS and filtering through a sterile 0.22 μm pore size PES membrane filter (Corning) or a sterile 0.2 μm pore size Supor PES syringe filter (Acrodisc, Pall), respectively. All freshly monomerized and diluted AGelD187N batches were diluted further with PBS in 1.5 ml Eppendorf tubes and heparin and ThT were added in respective order, each time mixing well by pipetting up and down several times. The final concentrations in the reaction mixtures were 5–28 μM of AGelD187N, 10 μM heparin and 50 μM ThT. The reaction mixtures were dispensed into 96 area plates (ref. 655 096, Greiner Bio-one) in 3–4 200 μl replicates and later into non-protein binding half area 96 well plates (ref. 3881, Corning) in 3–4 100 μl replicates. Seeding was done by adding 0.3–0.6 μl freeze-thawed preformed fibrils in each well or by dipping a thread (copolyamide monofilament, 0.46 mm, BASF) first in the preformed fibrils and then onto each well to be seeded. The plate was sealed with sealing film (LightCycler 480, Roche) as quickly as possible after seeding. Kinetic measurements were monitored at 37°C in Envision 2105 multimode plate reader (PerkinElmer) by measuring ThT fluorescence at 485 nm (20 nm bandwidth) upon excitation at 440 nm (20 nm bandwidth) every 10 minutes for 16–62 hours. Orbital shaking at 200 rpm (diameter 3 mm) for 15 s was performed before each measurement.

### Statistical analysis

GraphPad Prism software (v. 9.0) was used for data analyses. Maximum fluorescence intensities/maximum signals are means of three replicate reactions, except in the case of one high-signal 10 μM reaction (Fig 3A). The signal-to-background ratio (S/B) was calculated by dividing the mean maximum fluorescence intensity of the protein sample by the fluorescence intensity of the ThT control at the end of experiment [27]. The time to reach 50% of the maximum signal (t_50_) was calculated by fitting the kinetic data with a non-linear regression (five parameter) model until the timepoint when signal started declining.

### Transmission electron microscopy (TEM)

The carbon-coated copper grids (Formvar/carbon 200 mesh, prod.no. 01801, Ted Pella) were treated with glow discharge treatment (20 s) and an aliquot (4 μl) of each pooled well-mixed sample was placed on a separate grid. The samples were allowed to stand for 2 minutes on the grid before removing the excess solution with blotting paper. Each grid was washed twice with deionized water (4μl) and negatively stained for 1 min with 2% uranyl acetate in water (4 μl). The excess uranyl acetate was removed with blotting paper. The samples were air dried for at least 5 min under a petri dish cover. Transmission electron images were acquired using a JEM-1400 Plus transmission electron microscope with an acceleration voltage of 120 kV.

### Soluble aggregate analysis

After the aggregation assay, fibrils and insoluble aggregates were removed from the reaction mixtures by centrifuging the pooled replicates at 20 000 x g at +4°C for 1 hour. The separated supernatants (100 μl) were analysed for soluble aggregates and oligomers with SEC method described earlier.

### Characterization of monomerized polypeptide by SDS-PAGE, SEC-MALS and LC-MS

The monomerized polypeptide was analysed with precast Tris-tricine polyacrylamide gel (SDS-PAGE), with a gel percentage of 10-20% (4563113, Mini-Protean, Bio-Rad). A peptide analysis buffer was diluted from 10x stock (1610774, Bio-Rad). Five μl of the sample was mixed with 5 μl of the Tricine sample buffer (1610739, Bio-Rad) and 10 μl was loaded into the gel wells. A mixture of 12 recombinant proteins of 2–250 kDa (1610377, Bio-Rad) was used as a protein standard. Electrophoresis was performed in a Mini-Protean Tetra cell with an initial voltage of 50 V for 100 min, then 75 V for 60 min and 100 V for another 60 min. After the run, the bands were fixed by incubating the gel in a fixation solution of 40% methanol and 10% acetic acid for 30 minutes. The gel was stained with Imperial protein stain (24615, Thermo-Fisher) and imaged with a GelDoc EZ System (Bio-Rad).

The monomerized polypeptide was analysed with a SEC coupled with multiangle light scattering (SEC-MALS). Analytical SEC was performed using Advance Bio SEC 4.6 mm x 300 mm column (130 Å pore size, 2.7 μM particle size, Agilent) combined with an Advance Bio SEC 4.6 mm x 50 mm guard column (130 Å pore size, 2.7 μM particle size, Agilent). Isocratic elution was performed with a mobile phase (150 mM sodium phosphate buffer, pH 7.0) flow rate of 0.35 ml/min. The column was connected to a bioinert HPLC system (1260 Infinity II, Agilent) sequentially with a diode array detector (DAD WR, 1260 Infinity II, Agilent), a MALS detector (MiniDawn, Wyatt) and a refractive index (RI) detector (Optilab, Wyatt). The MALS detector employed a laser source at 659 nm and three detectors at different angles. RI detection was at 658 nm. Output signals from the DAD, MALS and RI detectors were imported into ASTRA software (Wyatt) for data processing. A bovine serum albumin standard (Wyatt) was employed as an independent control for molecular weight.

The monomerized polypeptide was analyzed with RP-HPLC coupled to a mass spectrometer (MS). A Thermo Fisher Scientific Vanguish LC system was fitted with a 2.1 mm BioZen XB-C8 intact 3.6 uM column (Phenomenex) heated to 60°C. The mobile phase B was 0.1% formic acid in acetonitrile while the mobile phase A was 0.1% formic acid. The gradient was 0 min 5% B; 2 min 5% B; 25 min 65% B; 30 min 90% B; 34 min 90% B; 35 min 5% B; 45 min 5% B. The flow rate was 0.25 ml/min. Samples were diluted to 5 μM concentrations in a 20 mM phosphate 1 M NaCl buffer (pH 7) and loaded in 10 μL quantities using the microliter pickup injection method. Samples injected into the column were analyzed by a Q Exactive (QE) mass spectrometer (Thermo Fisher Scientific) fitted with a HESI II Ion Max source (Thermo Fisher Scientific). Mass spectra were acquired at a resolution setting of 35,000 with a scan range from m/z 500 to 2,000 and settings including a spray voltage of 3.5 kV, a sheath-gas flow rate of 45, an auxiliary-gas flow rate of 10, an in-source collision-induced-dissociation (SID) value of 0 eV, and a capillary temperature of 275°C. Ion transfer optics throughout the instrument were tuned during annual maintenance and remained unchanged. Mass spectra were viewed and evaluated in Thermo Xcalibur Qual Browser software (Thermo Fisher Scientific) and processed using the Intact Protein Analysis module of BioPharma Finder 3.0. The default settings of the ReSpect deconvolution algorithm were initially used, with a subsequent adjustment for targeted mass and charge state ranges. Deconvolution mass tolerance of 50 ppm was used to identify individual charge states and their overall distribution.

## Results

### Purity and N-terminal modification affect the aggregation tendency of synthetic AGelD187N

We investigated three different variants of the synthetic AGelD187N polypeptide in this study (Table 1). First two variants had different RP-HPLC purities (95% and 90%) and the third variant had an N-terminal acetylation, which is commonly added to synthetic peptides to enhance their biological activity. The N-terminally acetylated variant had 95% purity. All three polypeptides were sonicated in 6 M guanidine hydrochloride and monomerized by gel filtration chromatography. The monomerization yields of different AGelD187N polypeptides were from 10–30% (S1 Table). Immediately after monomerization, polypeptides were diluted and used at a 25 μM concentration in the ThT based aggregation assay. The large enhancement of its fluorescence emission upon fibril binding makes ThT an unusually sensitive and efficient reporter [28]. However, different fibril structures and morphologies may elicit different level of fluorescence intensity enhancement [28–31]. ThT binding and fluorescence may also be affected by selected assay conditions, additives and molecules that are tested as aggregation inhibitors [32–34]. Therefore, caution is needed when interpreting the ThT fluorescence signals. In our assay, 10 μM heparin was constantly used to mimic the chemical environment of the extracellular matrix where the AGel amyloid deposits are observed in patients [13,35]. TEM and SEC analyses were used to verify the results of the ThT assay.

**Fig 2:**
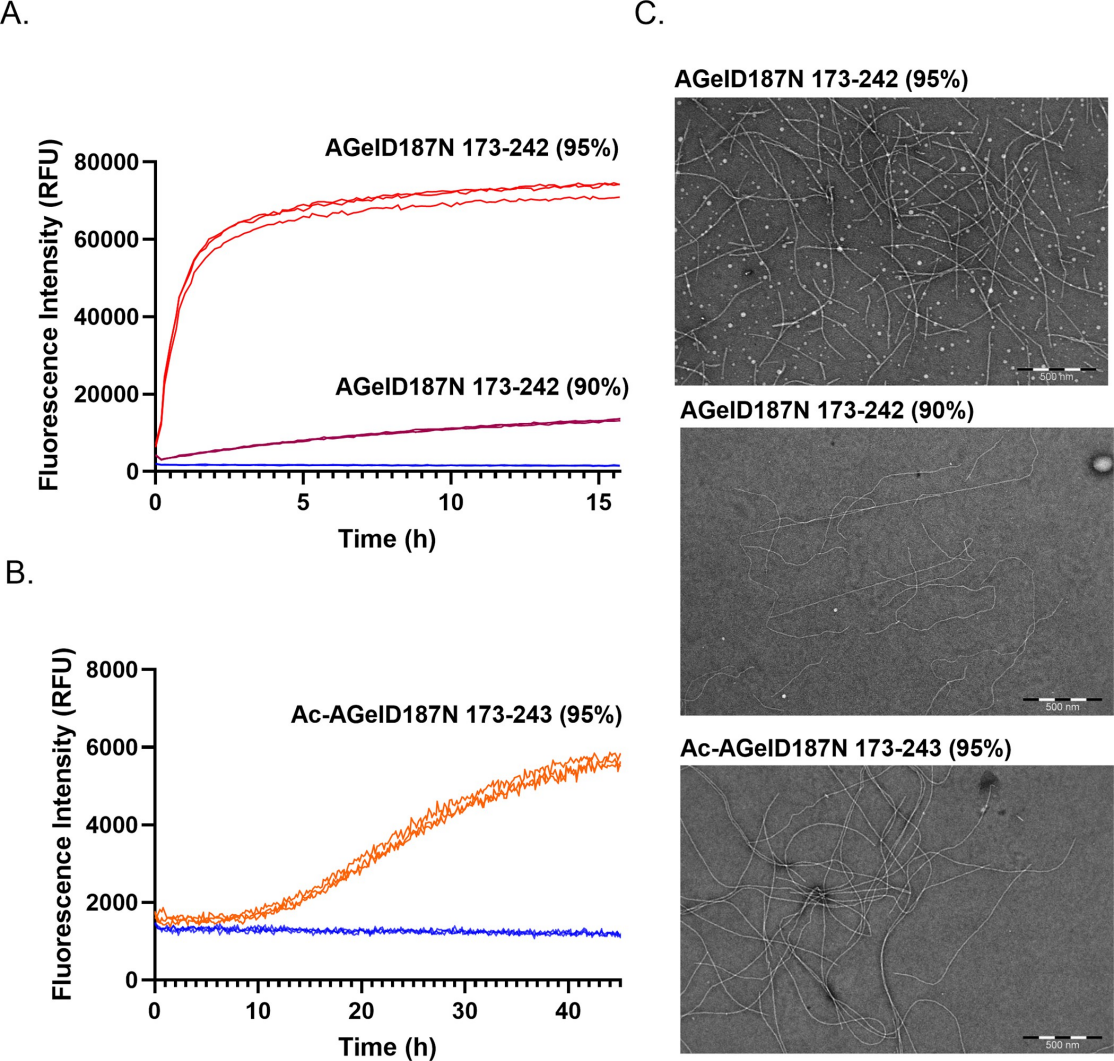
Amyloid formation of different synthetic AGelD187N polypeptides. (A) Amyloid formation kinetics of AGelD187N 173–242 with a 95% purity (red), AGelD187N 173–242 with a 90% purity (purple) and a ThT control (blue) monitored continuously by ThT fluorescence. Three replicate kinetic traces are shown. (B) Amyloid formation kinetics of Ac-AGelD187N 173–243 with a 95% purity (orange) and a ThT control (blue) monitored continuously by ThT fluorescence. Three replicate kinetic traces are shown. (C) Representative electron micrograph of each polypeptide after the aggregation experiment.

The 95% pure variant reached a maximum signal of 73 000 relative fluorescence units (RFU) in the aggregation assay whereas the 90% pure variant reached a maximum signal of only 13 000 RFU (Fig 2A). Corresponding S/B ratios were 37 and 7. This highlighted the importance of polypeptide purity in the aggregation experiments. The N-terminally acetylated 95% pure variant had only a modest maximum signal of 5800 RFU and S/B of only 4 (Fig 2B) indicating that the N-terminal acetylation had even greater negative effect on the aggregation tendency than low purity. The results of aggregation assay were validated with TEM. All synthetic polypeptides were able to form the same kind of amyloid fibrils (Fig 2C, S2 File). Micrographs revealed long, curvy, unbranching fibrils that were 7–8 nm in diameter; these are typical to amyloid fibrils [36,37] and recombinant AGelD187N 173–243 [35]. After these results were obtained, we continued our experiments using only non-acetylated >95% pure AGelD187N 173–242 (AGelD187N 173–242 (95%)).

### Initial aggregates affect the aggregation mechanism of synthetic AGelD187N 173–242

**Fig 3:**
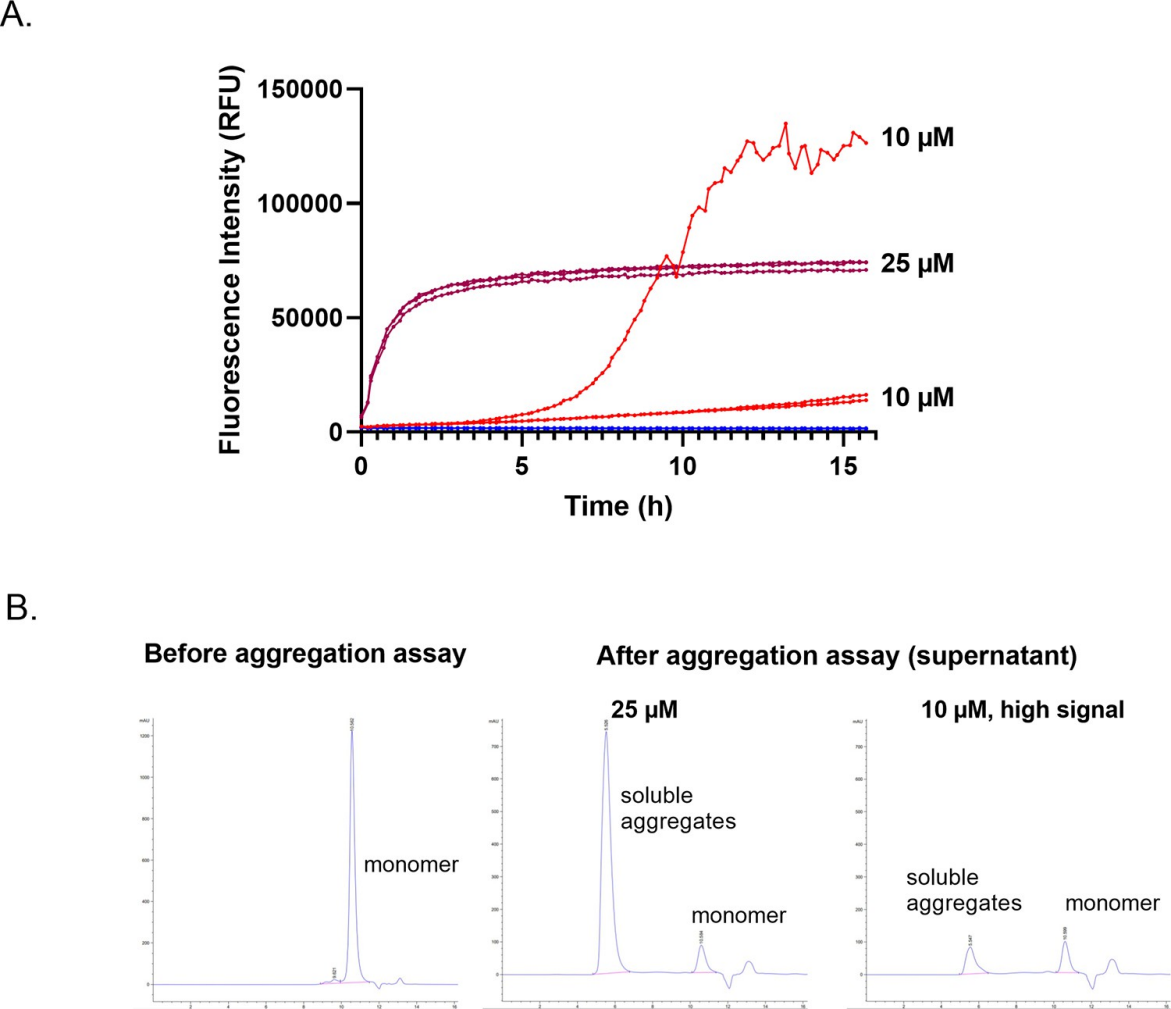
Lower concentration of synthetic AGelD187N 173–242 provokes sigmoidal aggregation curve and a more complete amyloid formation. (A) Amyloid formation kinetics of AGelD187N 173–242 at 10 μM (one high-signal and two low-signal reactions in red), at 25 μM (three reactions in purple) and the ThT control (three reactions in blue) monitored continuously by ThT fluorescence. (B) Analytical SEC chromatograms before the aggregation assay (left), after the aggregation assay from the assay supernatant of 25 μM reactions (middle) and from the assay supernatant of the high-signal 10 μM reaction (right).

The concentration of monomeric amyloidogenic protein must be high enough in the aggregation assay to display the nucleation and aggregate growth on laboratory time scale. However, low concentration would be more *in vivo* relevant and save precious material. In the first aggregation experiments, the synthetic AGelD187N polypeptides were studied at a 25 μM concentration. Next, AGelD187N 173–242 was studied at a 10 μM concentration by adding an extra dilution step to the preparation of the reaction mixture. As in the 25 μM reactions the signal had started from 6600 RFU indicating that there still were some initial ThT binding aggregates present in the reaction, in 10 μM reactions, the signal started from the background level of 2200 RFU indicating that there were no initial aggregates. In two of three replicate 10 μM reactions, the lag phase was still ongoing when the experiment was terminated (Fig 3A, bottom red curves). In the third replicate reaction, a sigmoidal kinetic trace was observed. The fluorescence signal started to rise steeply after a lag phase of five hours, and after an exponential phase, the maximum signal for this high-signal reaction was 130 000 RFU and the S/B 65 (Fig 3A, top red curve). As the maximum signal in the high-signal reaction was almost twice that of the 25 μM reactions (Fig 3A, purple curves), we concluded that the amyloid formation in the 25 μM reactions was incomplete. A SEC analysis from the assay supernatants supported this view as it revealed the high extent of soluble aggregates in the 25 μM reactions (Fig 3C, middle). In the high-signal 10 μM reaction there were far fewer soluble aggregates (Fig 3C, right).

### Seeding improves the reproducibility of synthetic AGelD187N 173–242 aggregation

**Fig 4:**
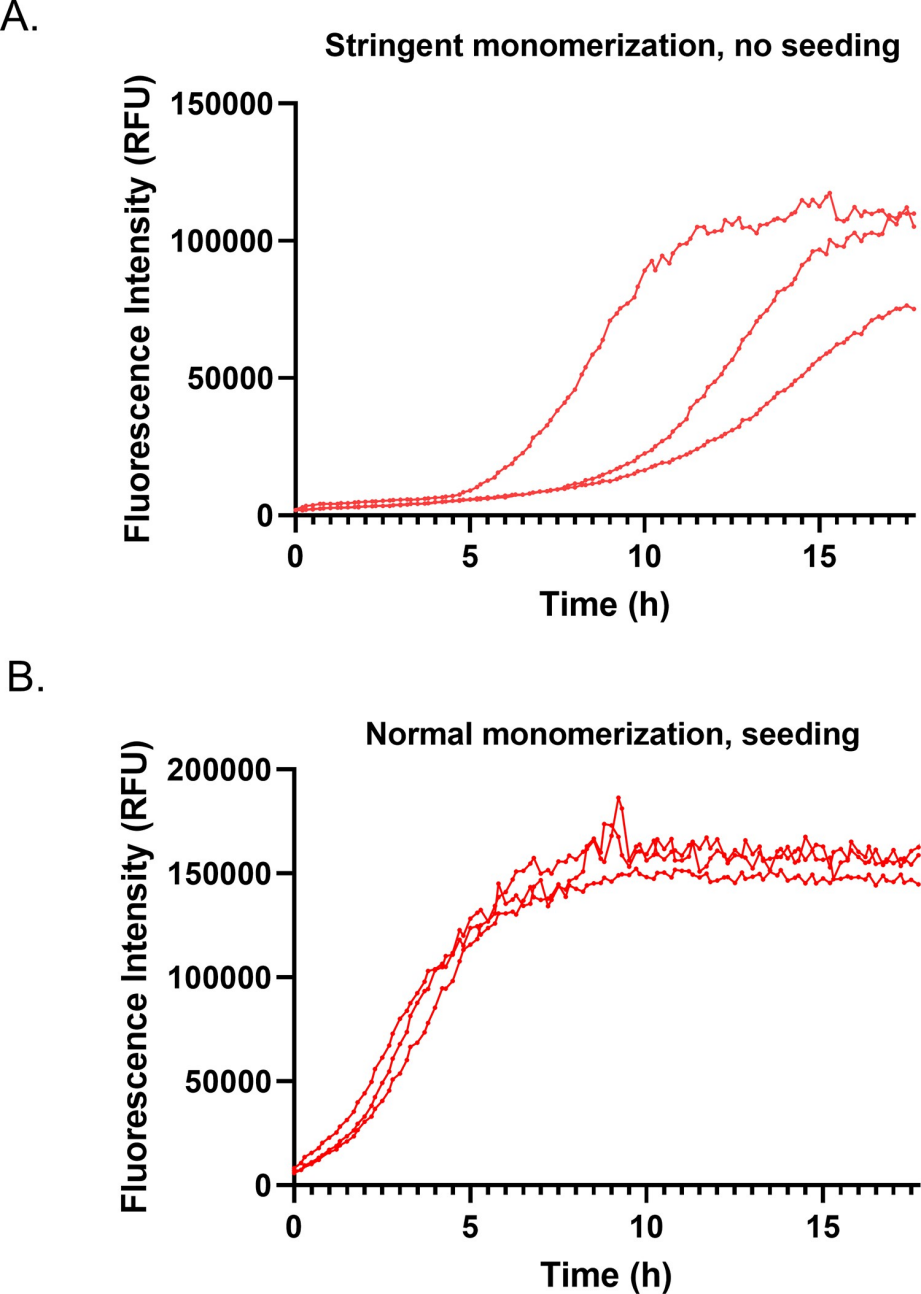
The effect of stringent monomerization and seeding on amyloid formation of synthetic AGelD187N 173–242. The effect of (A) stringent monomerization and (B) seeding (15 nM seeds) on amyloid formation of synthetic AGelD187N 173–242 at a 10 μM concentration monitored continuously by ThT fluorescence. Three replicate kinetic traces are shown.

In order to screen possible aggregation inhibitors, it is important that the amyloid protein aggregates in a repeatable manner in the aggregation assay. As previously described, a sigmoidal aggregation curve could be induced for synthetic AGelD187N 173–242 in just one of three replicate reactions. More stringent monomerization was tested to induce the exponential phase in all replicate wells in the aggregation assay. During monomerization, only one fraction from the middle of the main peak was collected instead of normal three main fractions. In the ThT assay, the exponential phase started in all the 10 μM reactions over the course of the experiment but at different timepoints (Fig 4A). The time required to reach 50% of the maximum signal (t_50_) varied from 8.5 h to 13.5 h. The average time was 11.5 ± 2.7 h. The maximum signal was 100 000 ± 22 000 RFU and the S/B was 50. These results demonstrated that even a slight difference in reaction mixture composition or conditions (i.e. an air bubble) can affect the sensitive aggregation process of a metastable solution [38].

To investigate whether synthetic AGelD187N 173–242 fibrils would have a seeding activity, 0.3 μl frozen and thawed reaction solution from the earlier experiments with sigmoidal curves was added to each reaction at the beginning of the experiment. Assuming that the preformed fibrils were uniformly resuspended after plate shaking, the final concentration of the preformed fibrils in each reaction solution was 15 nM. Seeding practically eliminated the lag phase observed in the spontaneous conversion and almost doubled the maximum signal (Fig 4B). The t_50_ was 3.7 ± 0.4 h and the maximum signal 170 000 ± 17 000 RFU. The S/B was improved to 85. As a result of the seeding, the reproducibility of the replicate measurements was improved to enable the screening of potential inhibitors.

### Aggregation assay down-scaling reduces the synthetic AGelD187N 173–242 demand

**Fig 5:**
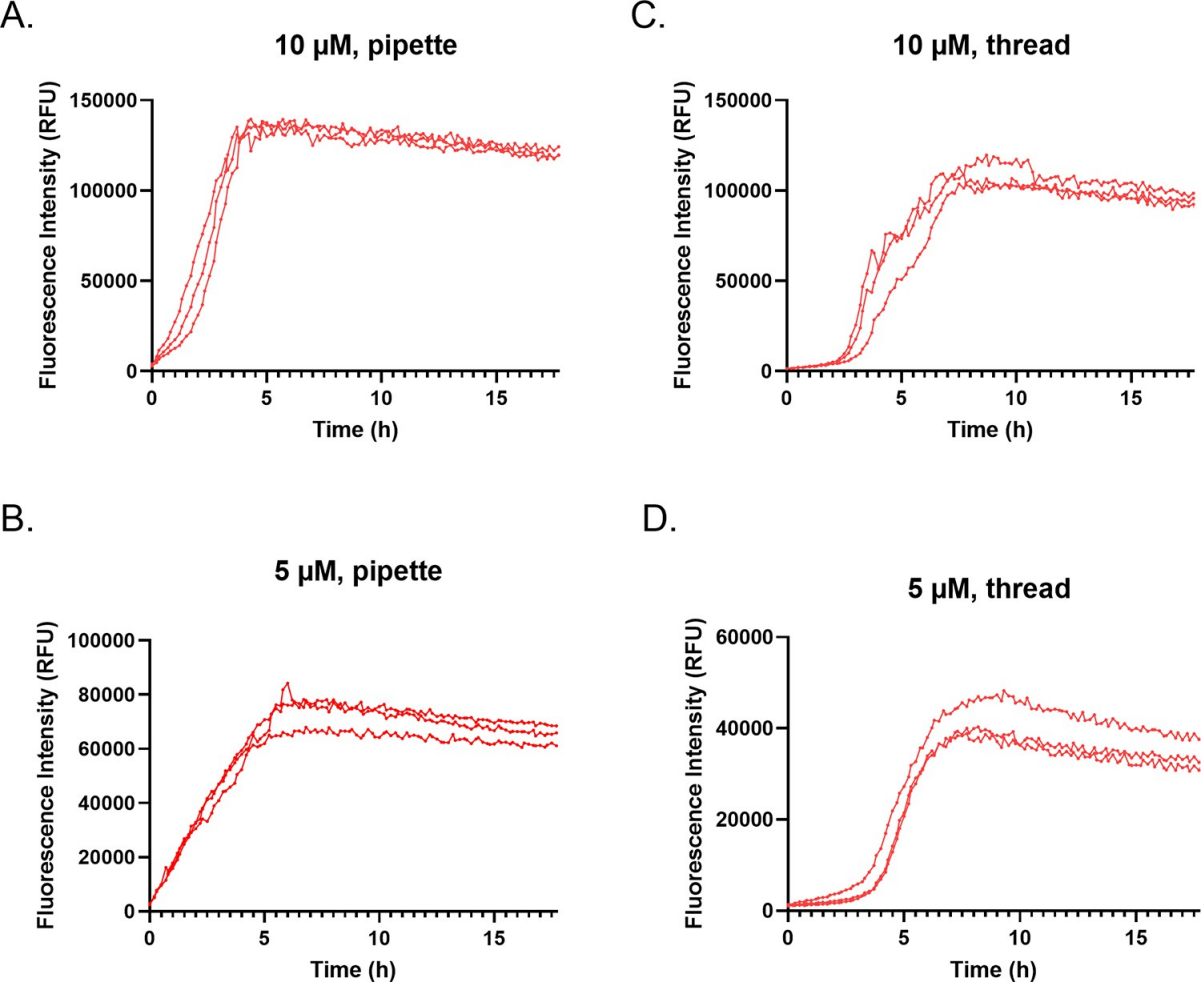
Synthetic AGelD187N 173–242 aggregation assay optimization. Amyloid formation of synthetic AGelD187N 173–242 in 100 μl reaction volume at (A) 10 μM, pipette seeded with 30 nM seeds, (B) 5 μM, pipette seeded with 30 nM seeds, (C) 10 μM with thread seeding, and (D) 5 μM with thread seeding, monitored continuously by ThT fluorescence. Three replicate kinetic traces are shown.

To diminish the polypeptide consumption, the seeded aggregation reaction was transferred to half-area plates (reaction volume 100 μl). Synthetic AGelD187N 173–242 was studied at 10 μM and 5 μM concentrations. At a 10 μM concentration, the t_50_ was 2.4 ± 0.3 h and the maximum signal was 140 000 ± 2000 RFU (Fig 5A). The S/B was 127. The aggregation rate and S/B ratio were higher than in 200 μl reaction volume. This was logical as the relative ratio of seeds to monomers was higher (30 nM to 10 μM). At a 5 μM concentration, the t_50_ was 2.4 ± 0.03 h and the maximum signal was around half of the signal of the 10 μM concentration, 77 000 ± 8200 RFU (Fig 5B). The S/B was 70. The lag phase was fully bypassed since the relative ratio of seeds to monomers was even higher (30 nM to 5 μM). At both concentrations, the aggregation curves were reproducible.

To diminish the number of transferred seed, a thread seeding technique was tested. We dipped a thin copolyamide thread first to the undiluted preformed fibrils and then to the well with monomers. As expected, the lag phase was longer compared to pipette seeding due to the lower number of transferred seeds (Fig 5C and 5D). At 10 μM, aggregation rates started to increase exponentially two hours after starting the experiment (Fig 5C). The t_50_ was 5.4 ± 1.9 h. The maximum signal was 110 000 ± 7000 RFU. The S/B was 100. At 5 μM, the aggregation rates started to increase exponentially three hours after starting the experiment (Fig 5D). The t_50_ was 5.3 ± 0.4 h. The maximum signal was 42 000 ± 5000 RFU and the S/B was 39. Although thread seeding was successful in triggering aggregation in all wells, the reproducibility of the aggregation was not as good as with pipette seeding.

### Synthetic AGelD187N 173–242 is monomeric and pure after the monomerization

Finally, we investigated more thoroughly the quality of synthetic AGelD187N 173–242 monomerized with normal monomerization protocol and used successfully in seeded aggregation experiments. This was important in order to establish satisfactory criteria for the synthetic monomerized material to be used in future aggregation experiments.

**Fig 6:**
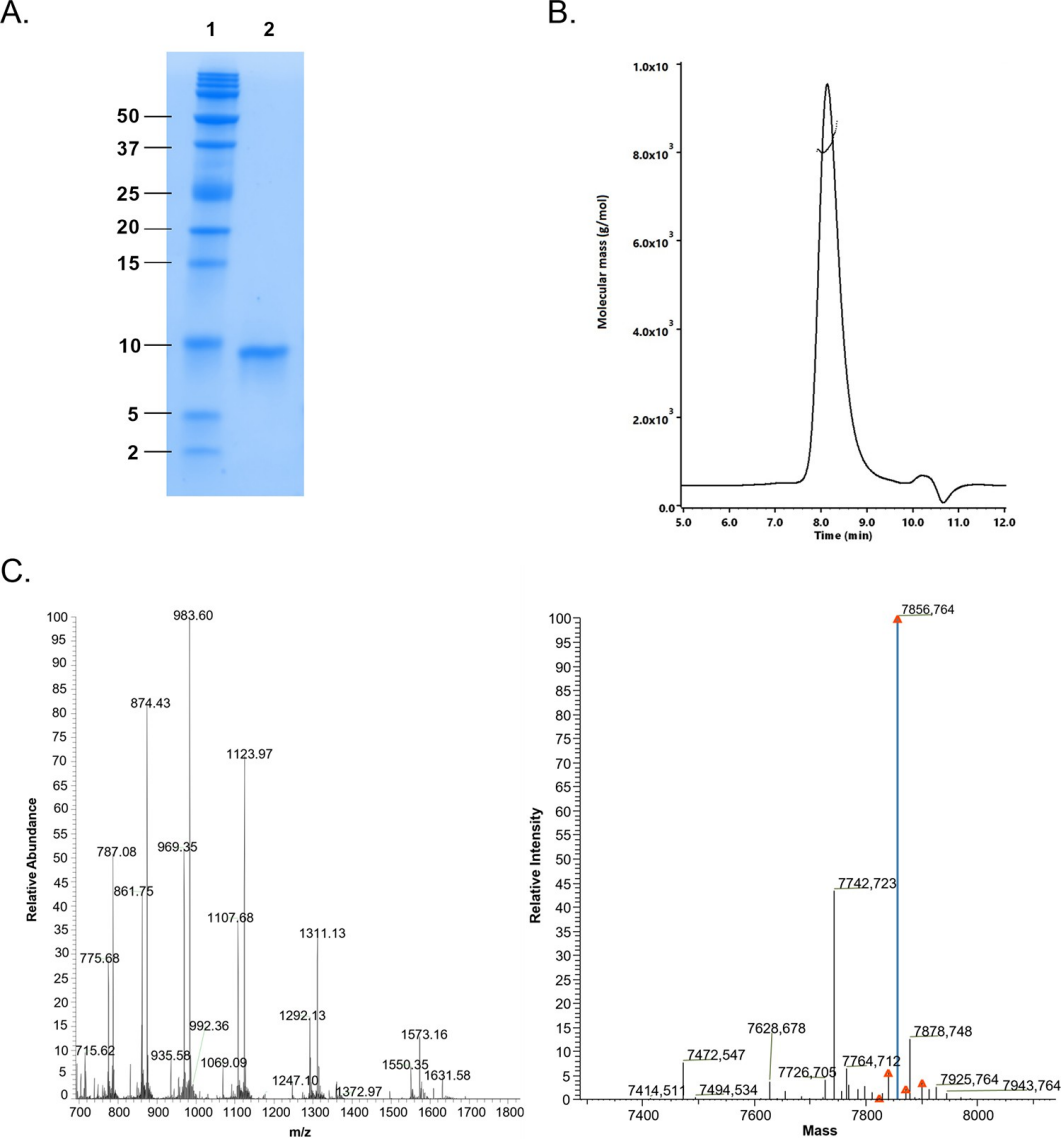
Purity assessment of synthetic AGelD187N 173–242. Purity assessment of synthetic AGelD187N 173–242 after monomerization (A) on Tricine-SDS-PAGE gel including molecular weight standard (Lane 1) and AGelD187N 173–242 (Lane 2), (B) SEC-MALS analysis and (C) LC-MS analysis; mass spectrum (left) and deconvolved spectrum (right).

In the SDS-PAGE analysis with Tris-Tricine gel (suitable for small proteins) a clear band was visible at the location of 8 kDa for monomerized synthetic AGelD187N 173–242 (Fig 6A, S1 Raw images). According to the SEC-MALS analysis, monomerized synthetic AGelD187N 173–242 did not contain aggregates or fragments and the molar mass was 8 kDa (Fig 6B). According to LC-MS analysis, the measured monoisotopic mass 7857.8 Da correspond fully to the calculated monoisotopic mass of AGelD187N 173–242 (7857.8 Da) (Fig 6C).

## Discussion

In this work we have described a practical and drug discovery suitable approach to study *in vitro* the aggregation kinetics of the main amyloidogenic polypeptide in Finnish gelsolin amyloidosis, the 70-residue gelsolin fragment AGelD187N 173–242. We have been able to show that synthetic AGelD187N 173–242, monomerized in a similar way to recombinant AGelD187N 173–242, is able to form fibrils typical of amyloid diseases. Reproducible aggregation kinetics are attainable with this full-length synthetic material when correctly handled. Earlier studies with shorter synthetic stretches of AGelD187N 173–242 are less *in vivo* relevant, since shorter peptides do not have all the features the disease-related full-length fragment has. On the other hand, the biotechnological production of all amyloidogenic polypeptides is cumbersome, and extremely pure recombinant material with sufficient yields are difficult to obtain. The utilization of synthetic material enables large screening campaigns where considerable amounts of amyloidogenic protein is needed.

There are several crucial matters that need to be considered to succeed in *in vitro* aggregation assay development with synthetic material. The quality of starting material and its correct handling cannot be over emphasized, even though it is already raised in previous literature [11,12,38,39]. Broadening the RP-HPLC purity criteria from >95% to >90% had a drastic negative effect on the aggregation tendency of the polypeptide. Also, we discovered that the N-terminal acetylation, which is routinely added to synthetic peptides to protect them, significantly decreased the aggregation tendency of AGelD187N 173–242. This is probably due to the changed charge and hydrophobicity of the N-terminus. We suggest that in the case of aggregation assays, the addition of extra terminal modifications to synthetic polypeptides should be avoided.

It is very important to ensure that the starting material is truly monomeric when an aggregation experiment is started. For assays that rely on ThT dye, this can be done easily by checking that the initial fluorescence signal is on a background level at the beginning of the experiment. We ascertained that although we had performed a careful monomerization to our synthetic polypeptide, only after diluting the monomerization product to a 10 μM concentration, would the initial fluorescence signal come down to background level and a new nucleation and typical sigmoidal aggregation curve be provoked. Most likely the reversibly associated species still left after the monomerization dissociated back to monomers or small non-ThT binding oligomers when the equilibrium was shifted more to the direction of stable monomers [38]. A truly monomeric starting material results in clean mass balance, this means that most material will convert into fibrils and there will be less off-pathway aggregates that do not proceed into fibrils [40]. The analytical SEC results confirmed that when the aggregation curve was sigmoidal, there were only a few soluble aggregates present in the supernatant after the assay. In contrast, at a 25 μM concentration, when there were initial aggregates present at the beginning of the reaction and the aggregation curve was concave, there were a considerable amount of soluble high molecular weight aggregates present that had not proceeded into fibrils. Since growth of an aggregate occurs at higher rate with a lower free energy barrier than nucleation [38], most likely only the already existing off-pathways aggregates grew until the monomer concentration reached the solubility limit. This indicates that ´correct´ amyloid formation is not able to occur when the starting material is not fully homogenous monomer solution.

Primary nucleation is an intrinsically slow event characterized by high free energy barrier [38]. This causes high sensitivity to any variation in sample or environment and inevitable variation in the timescale of the aggregation process, as we experienced in our first experiments with a 10 μM concentration. By adding preformed AGelD187N 173–242 fibrils from earlier experiments with sigmoidal curves into the monomer solution at the beginning of the experiment, we achieved a reproducible on-pathway aggregation in all wells simultaneously. Other benefits of the seeded assay are higher S/B ratio, a shorter assay time and no requirement for a stringent monomerization protocol, which saves precious material. We established that the reproducibility of the seeded assay for synthetic AGelD187N 173-242 could be maintained when the reaction volumes were decreased from 200 μl to 100 μl and the polypeptide concentration from 10 μM to 5 μM. Down-scaling enables a higher number of possible inhibitors to be screened with the same amount of assay components. We were able to transfer seeds by thread seeding but due to its manual nature, the slowness and variation introduced, we suggest that thread seeding should not be used when screening potential inhibitors. Instead of this procedure, we recommend that automation is worth considering as a means of transferring very small amounts of preformed fibrils. By utilizing a liquid handling robot, the whole assay could be down-scaled further to a 384-well plate format.

The bottleneck in setting up *in vitro* aggregation assays for amyloid proteins has previously been to obtain enough amyloid proteins in a sequence homogenous and pure form. In addition, many amyloid proteins display very slow primary nucleation and are therefore highly sensitive to any variation in the solution and experimental conditions. Our study offers strategies to overcome these challenges. We have shown that a 70-residue-long polypeptide produced by chemical synthesis and monomerized by gel filtration can be used for reproducible aggregation follow-up when aggregation is triggered by seeding. The assay presented in this study now enables the screening of possible anti-aggregational hit compounds against gelsolin amyloidosis, a severe inherited disease currently with no cure. As chemical synthesis methods will constantly be improving generating amyloidogenic polypeptides at a faster rate, a greater purity, and a lower cost, we hope to see that our methodology could be successful with other misfolding proteins in the future.

## Supporting information

S1 Table Monomerization yields of different AGelD187N polypeptides. (PDF)

S1 Raw images Original gel image for Fig 6A. (PDF)

S1 File RP-HPLC and MS data of AGelD187N polypeptides before monomerization. (PDF)

S2 File Electron micrographs of AGelD187N polypeptides after the aggregation experiment. The original electron micrographs for Fig 2C and one higher magnification electron micrograph of each sample.
(PDF)
