# Supplementary material for: Development of an in vitro aggregation assay for long synthetic polypeptide, amyloidogenic gelsolin fragment AGelD187N 173–242
Source: PLoS One. 2023 Aug 17;18(8):e0290179. doi: 10.1371/journal.pone.0290179 (PMC10434866; doi:10.1371/journal.pone.0290179)
Supplement: S1 Table — (PDF) [file pone.0290179.s001.pdf]

Polypeptide	Monomerized amount (mg)	Yield (mg)	Yield (%)
AGelD187N 173-242 (95%)	1.0	0.3	30
AGelD187N 173-242 (90%)	12.7	1.3	10
Ac-AGelD187N 173-243 (95%)	3.0	0.5	17
