# Supplementary figures and images for: Development of an in vitro aggregation assay for long synthetic polypeptide, amyloidogenic gelsolin fragment AGelD187N 173–242
Source: PLoS One. 2023 Aug 17;18(8):e0290179. doi: 10.1371/journal.pone.0290179 (PMC10434866; doi:10.1371/journal.pone.0290179)

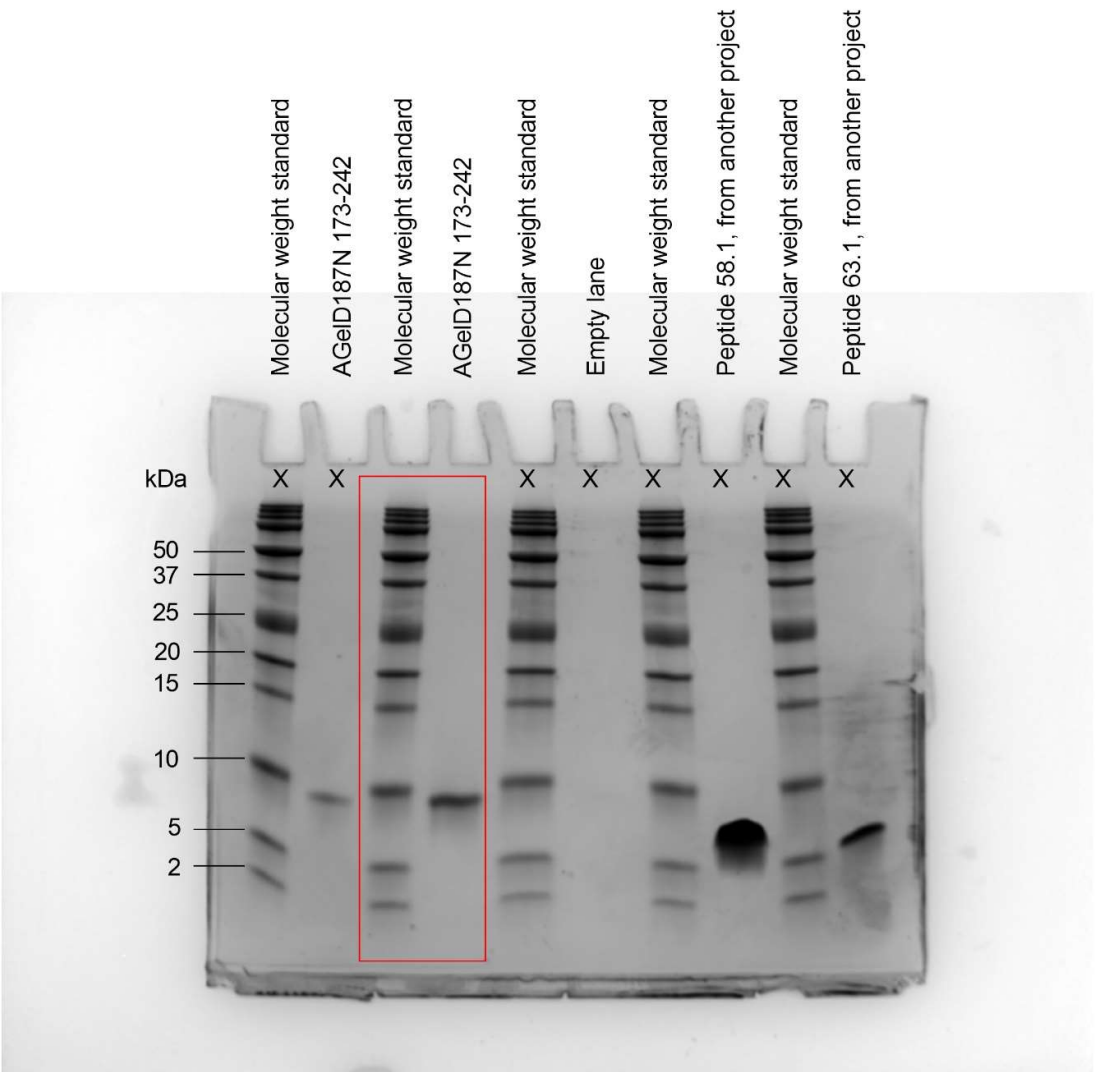

Supplement: S1 Raw images — (PDF) [file pone.0290179.s002.pdf]
