# Supplementary material for: Development of an in vitro aggregation assay for long synthetic polypeptide, amyloidogenic gelsolin fragment AGelD187N 173–242
Source: PLoS One. 2023 Aug 17;18(8):e0290179. doi: 10.1371/journal.pone.0290179 (PMC10434866; doi:10.1371/journal.pone.0290179)
Supplement: S1 File — (PDF) [file pone.0290179.s003.pdf]

AGelD187N 173-242 (95%): RP-HPLC

Column:	4.6 x 250mm, 5µm		
Solvent A	A: 0.1% Trifluoroacetic Acid in 100% Acetonitrile		
Solvent B	B: 0.1% Trifluoroacetic Acid in 100% Water		
Gradient:		A	B
	0.0min	20%	80%
	25.0min	60%	40%
	25.1min	100%	0%
	30.0min	Stop	
Volume:	5µl		
Wavelength:	220nm		
Flow rate:	1.0ml/min		

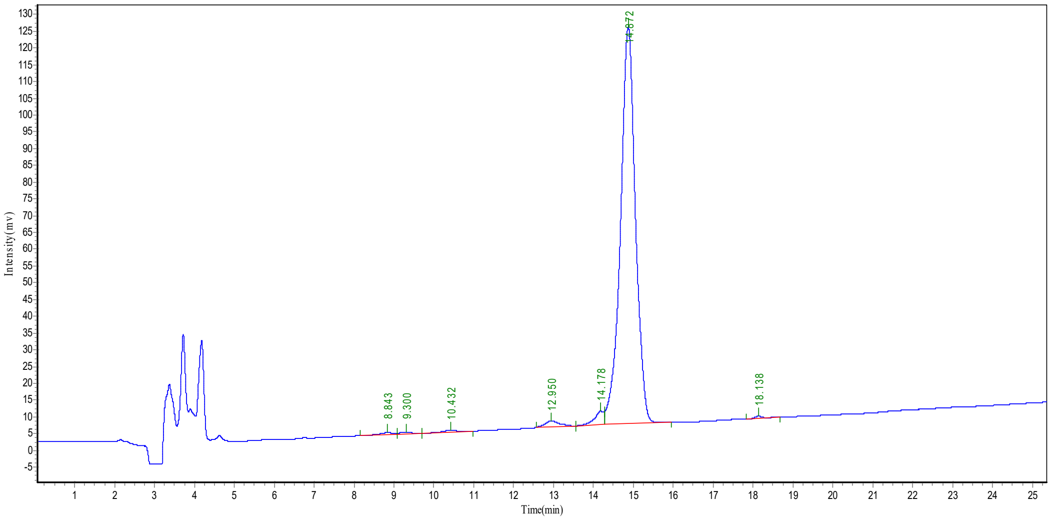

Peak	Time	Height	Area	Conc.
1	8.843	628.344	14963.770	0.4604
2	9.300	588.313	10810.620	0.3326
3	10.432	629.730	12814.859	0.3943
4	12.950	1671.408	41324.953	1.2714
5	14.178	4033.815	68577.414	2.1098
6	14.872	118403.875	3092357.750	95.1385
7	18.138	663.663	9526.306	0.2931
Total		100.0000		

AGelD187N 173-242 (95%): Mass Spectrometry

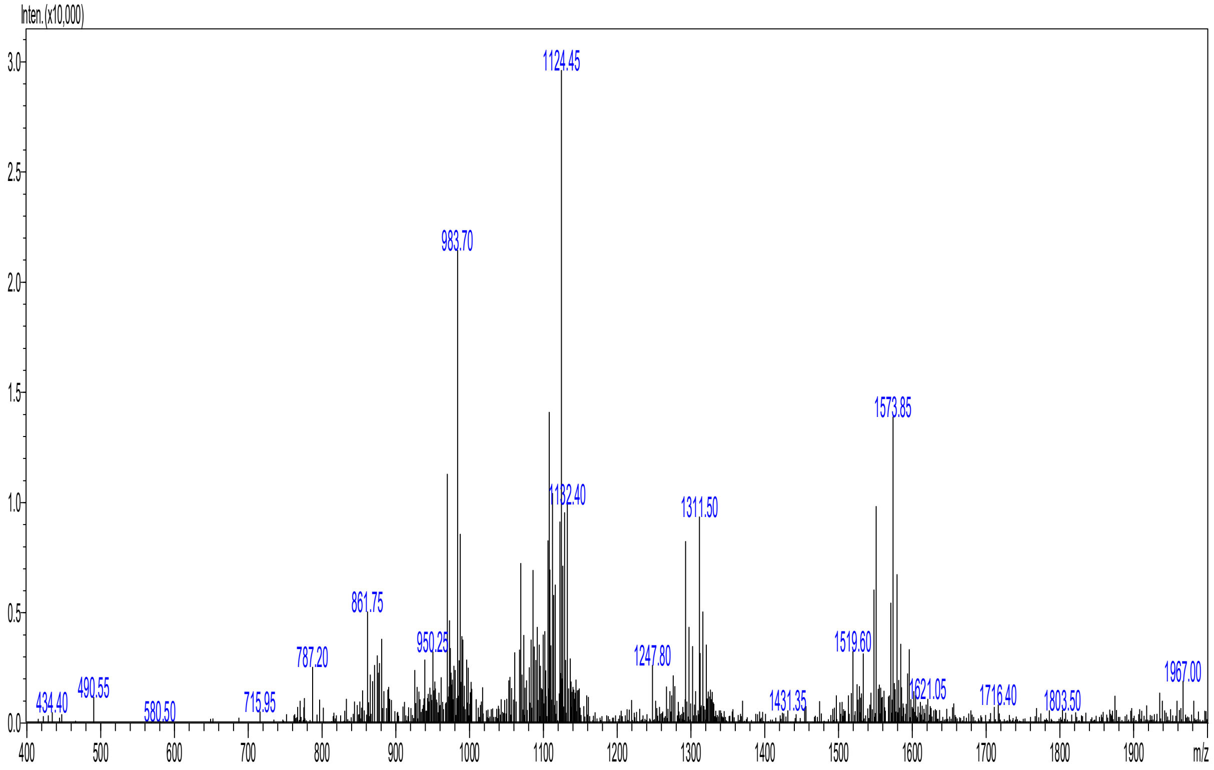

Method: ESI
Main Peak: 1124.45
MW [M+7H^{+}]: 7871.15
MW: 7864.15
Theoretical MW:7861.46
Match: Approved
Z=7+

AGelD187N 173-242 (90%): RP-HPLC

Column: 4.6 x 250mm, 5µm
Solvent A A: 0.1% Trifluoroacetic Acid in 100% Acetonitrile
Solvent B B: 0.1% Trifluoroacetic Acid in 100% Water
Gradient:

	A	B
0.0min	20%	70%
25.0min	60%	40%
25.1min	100%	0%
30.0min	Stop	

Volume: 10µl
Wavelength: 220nm
Flow rate: 1.0ml/min

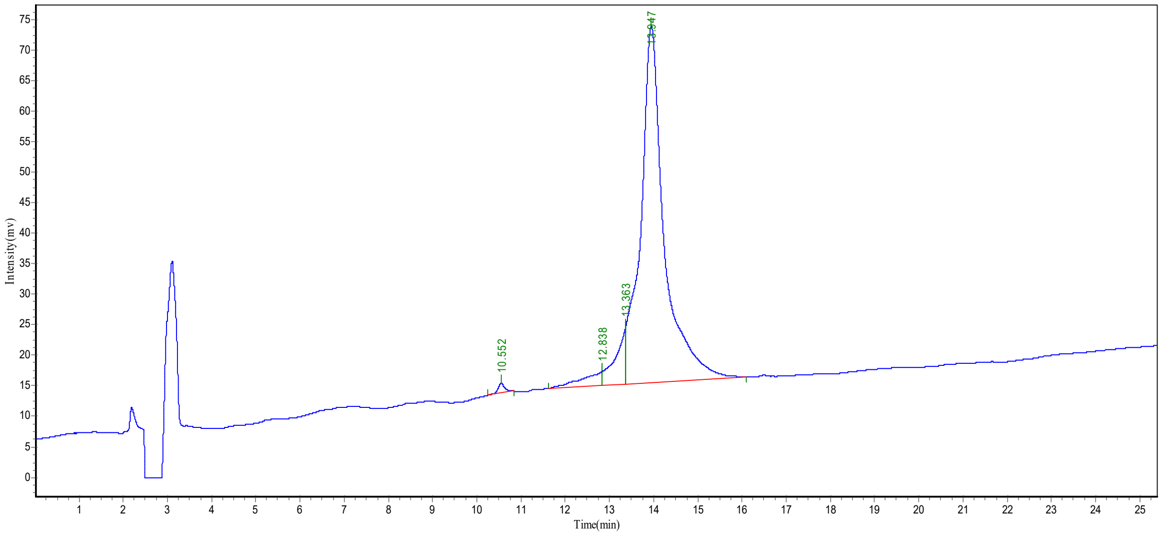

Peak	Time	Height	Area	Conc.
1	10.552	1599.474	17718.805	0.7425
2	12.838	2238.750	73222.602	3.0684
3	13.363	9179.171	142736.109	5.9813
4	13.947	58306.027	2152700.500	90.2079
Total		100.0000		

AGelD187N 173-242 (90%): Mass Spectrometry

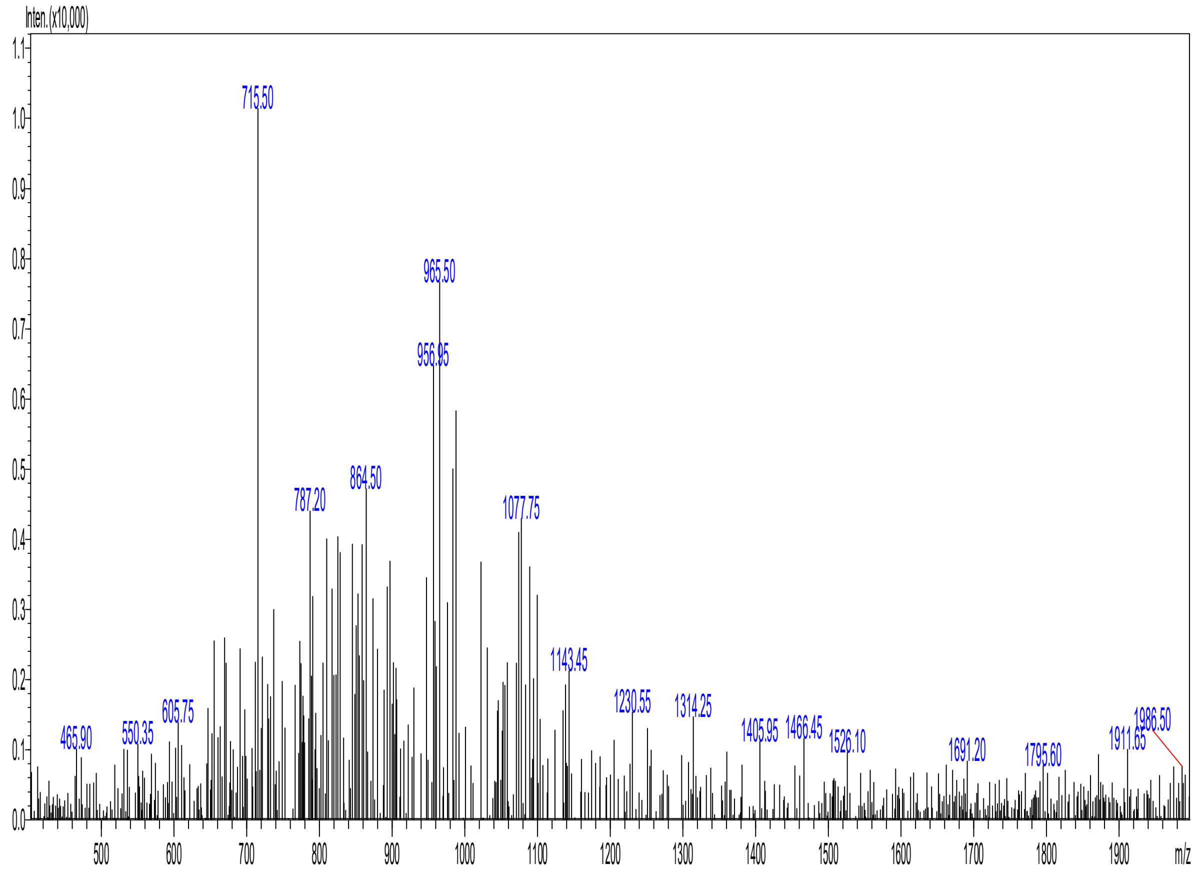

Method: ESI
Main Peak: 715.50
MW [M+11H$^+$]: 7870.5
MW: 7859.5
Theoretical MW:7861.46
Match: Approved
Z=11+

Ac-AGelD187N 173-243 (95%): RP-HPLC

Column:	4.6×250mm, C4 300A		
Solvent A	A: 0.1% Trifluoroacetic Acid in 100% Acetonitrile		
Solvent B	B: 0.1% Trifluoroacetic Acid in 100% Water		
Gradient:		A	5
	0.0min	10%	90%
	25.0min	100%	0%
	25.1min	100%	0%
	30.0min	Stop	
Volume:	5µl		
Wavelength:	214nm		
Flow rate:	1.0ml/min		

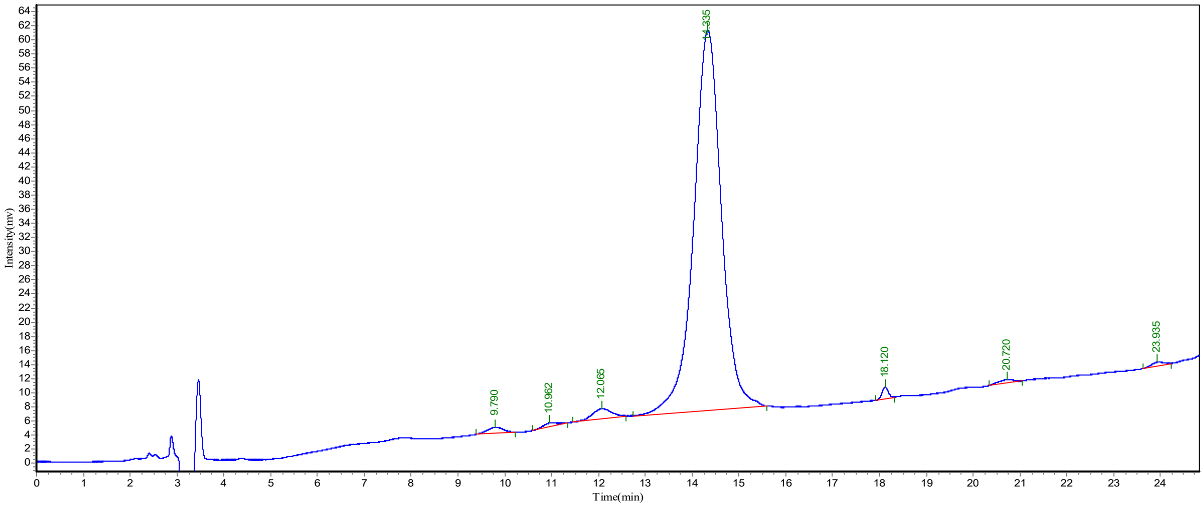

Rank	Time	Height	Area	Conc.
1	9.790	872.960	20931.203	0.9021
2	10.962	552.566	10518.392	0.4533
3	12.065	1429.959	39817.590	1.7161
4	14.335	53880.793	2212197.000	95.3455
5	18.120	1673.948	15343.996	0.6613
6	20.720	532.459	11348.769	0.4891
7	23.935	578.822	10033.604	0.4324
Total				100.0000

Ac-AGelD187N 173-243 (95%): Mass Spectrometry

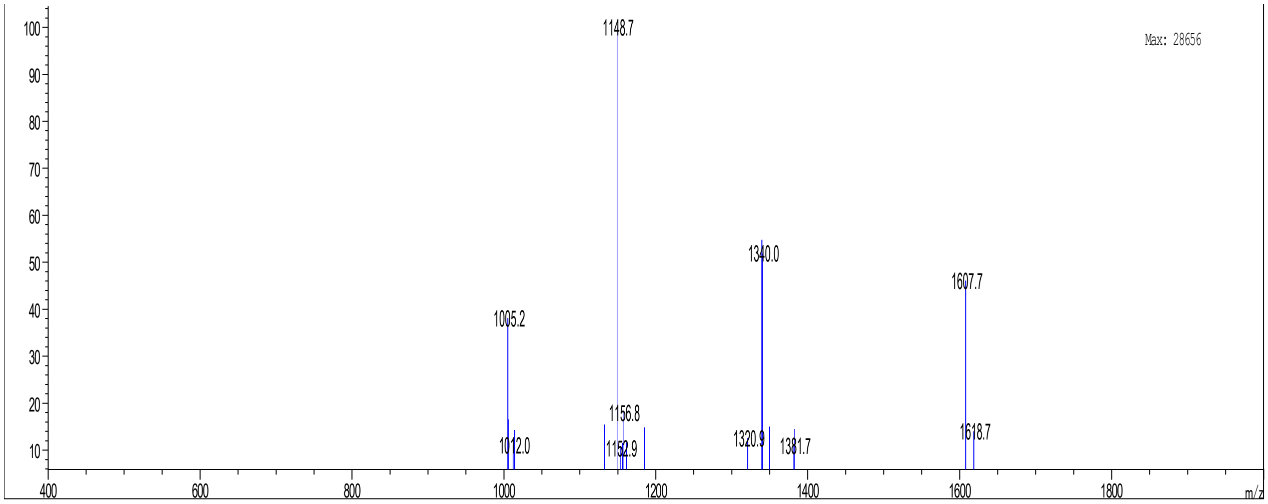

Method: ESI
Main Peak:1148.7
MW [M+7H$^+$]: 8040.9
MW: 8033.9
Theoretical MW: 8034.85
Match: Approved
Z=7+
