# Supplementary material for: Development of an in vitro aggregation assay for long synthetic polypeptide, amyloidogenic gelsolin fragment AGelD187N 173–242
Source: PLoS One. 2023 Aug 17;18(8):e0290179. doi: 10.1371/journal.pone.0290179 (PMC10434866; doi:10.1371/journal.pone.0290179)

AGelD187N 173-242 (95%), 20 000X. This image is in Fig 2C.

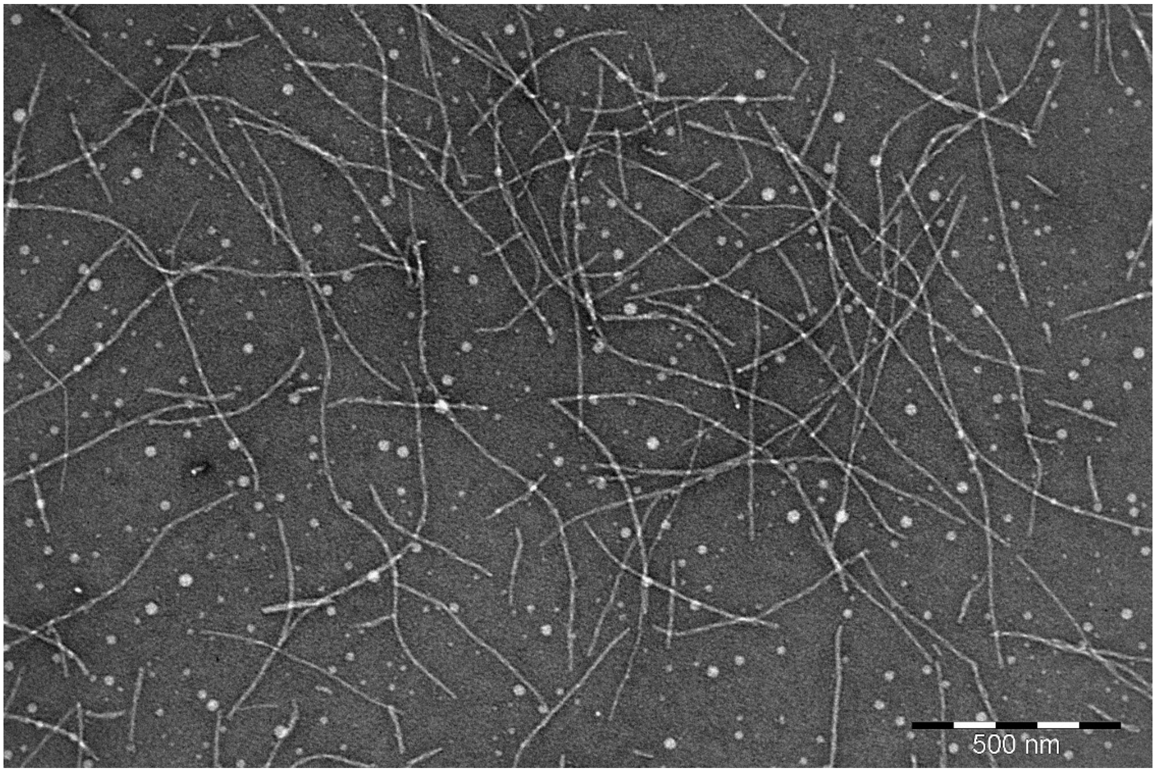

AGelD187N 173-242 (95%), 30 000X

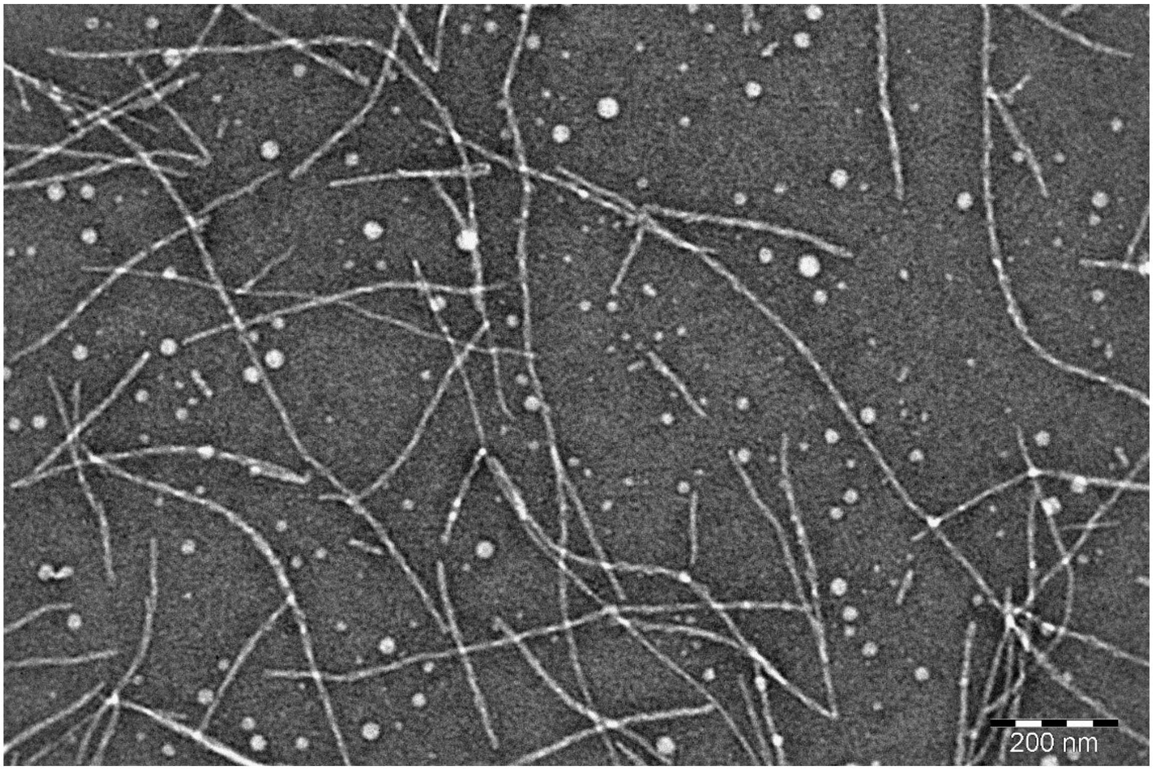

AGelD187N 173-242 (90%), 20 000X. This image is in Fig 2C.

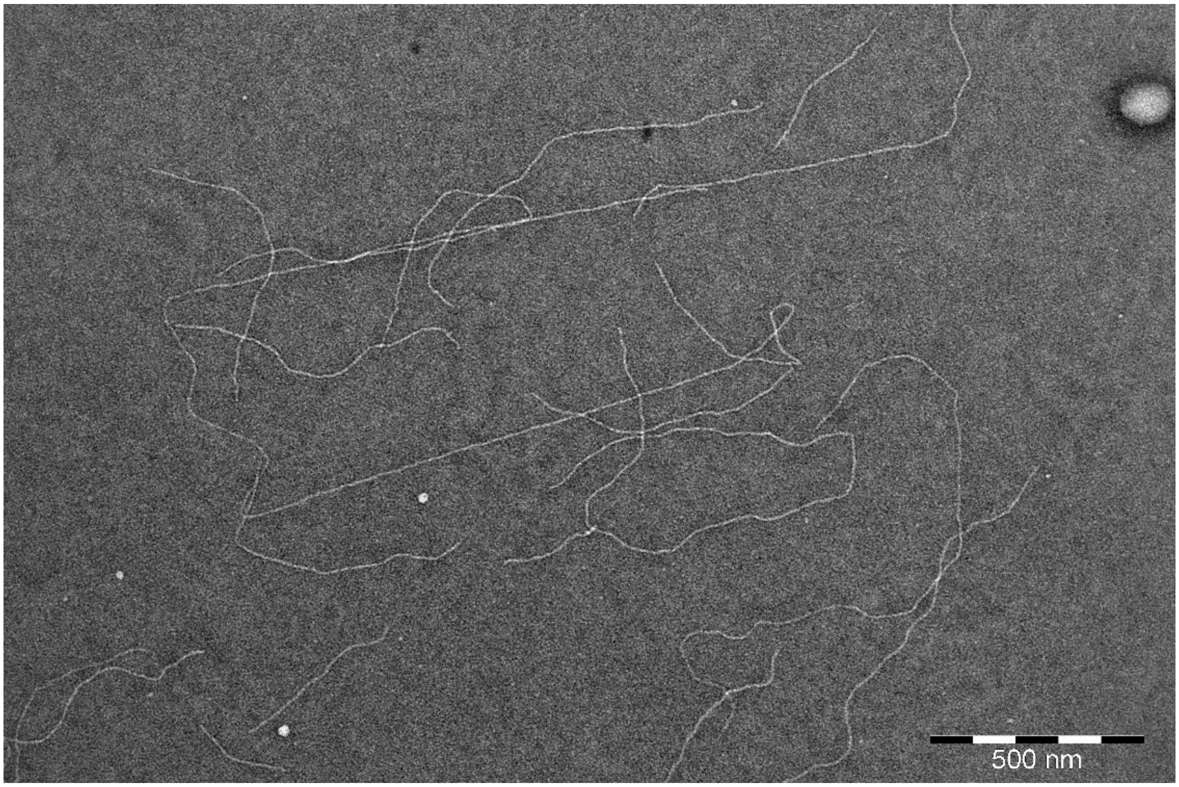

AGelD187N 173-242 (90%), 40 000X

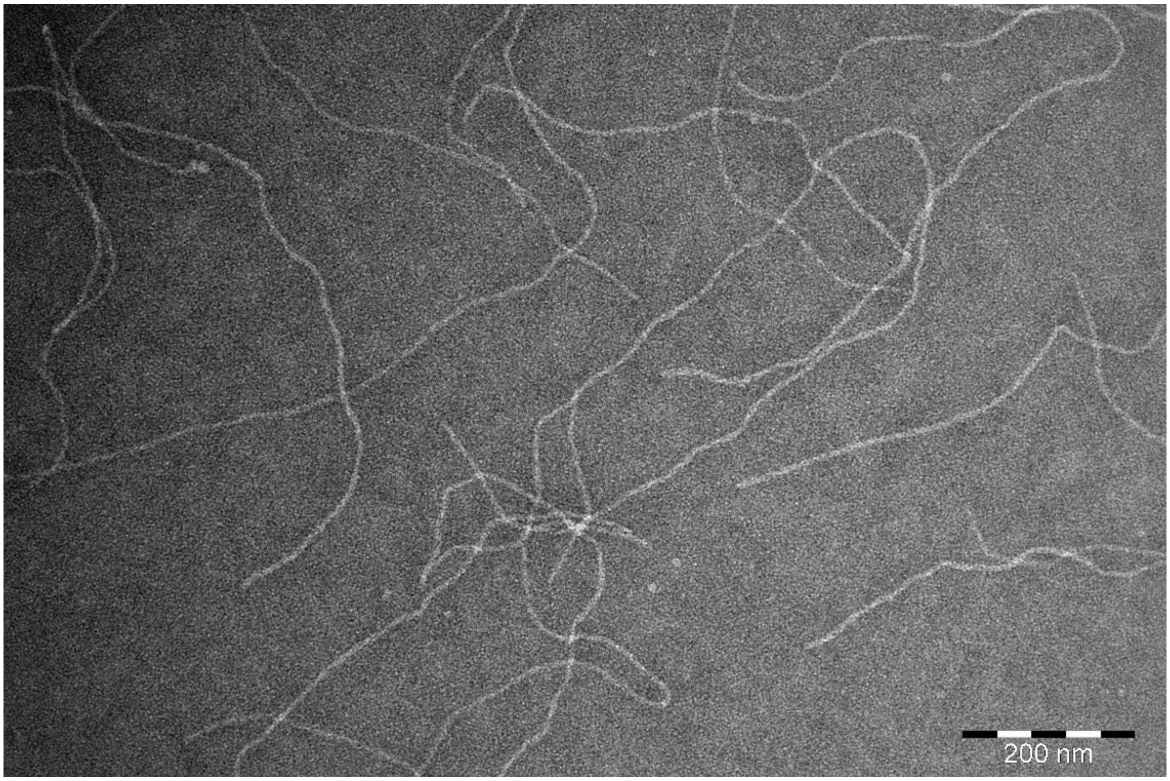

Ac-AGelD187N 173-243 (95%), 20 000X. This image is in Fig 2C.

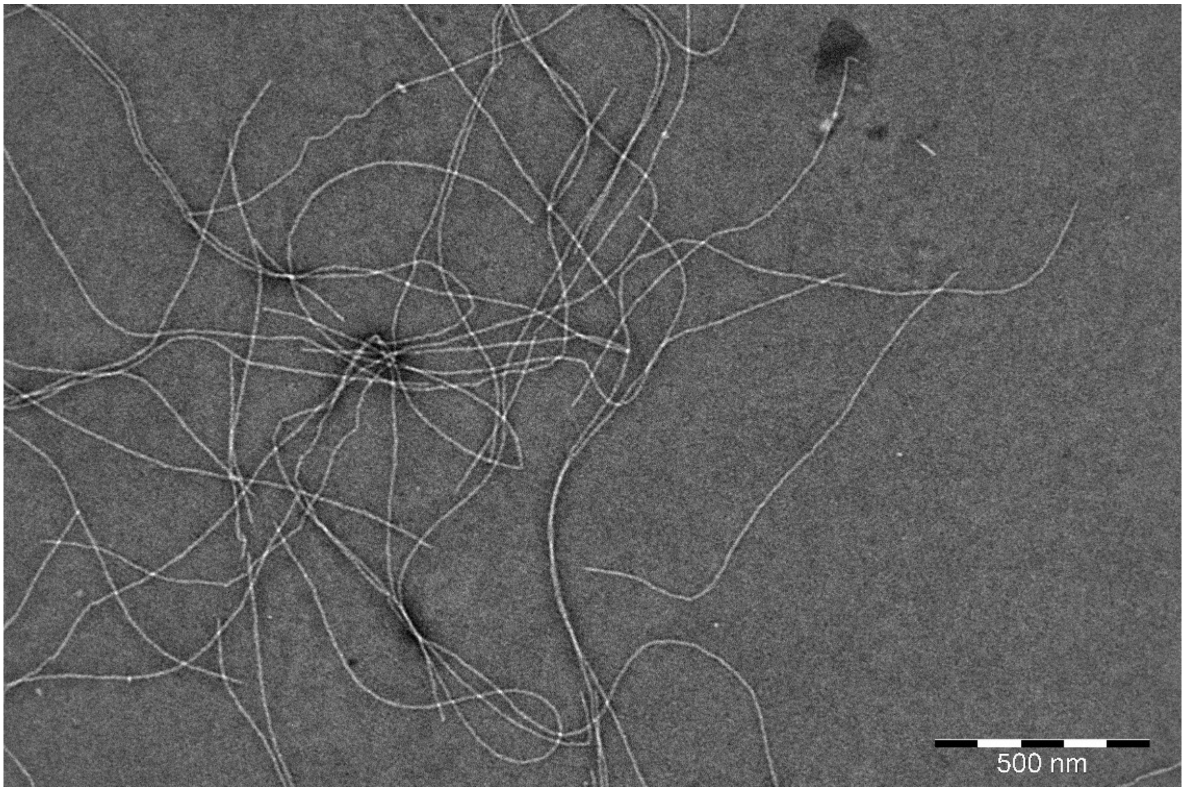

Ac-AGelD187N 173-243 (95%), 60 000X

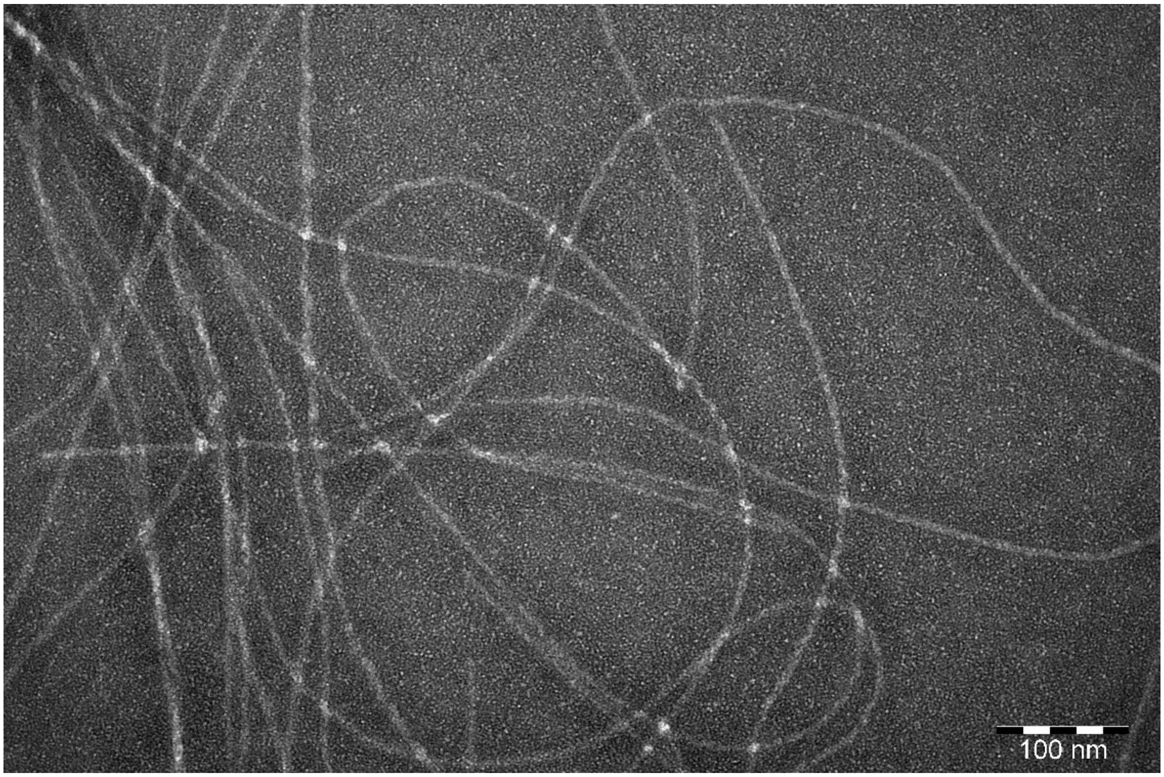

Supplement: S2 File — The original electron micrographs for Fig 2C and one higher magnification electron micrograph of each sample. (PDF) [file pone.0290179.s004.pdf]
